# Periodontal parameters of two types of 3 x 3 orthodontic retainer: a longitudinal study

**DOI:** 10.1590/2177-6709.24.3.064-070.oar

**Published:** 2019

**Authors:** Larissa A. Ferreira, Diogo M. Sapata, Maria G. A. Provenzano, Roberto M. Hayacibara, Adilson L. Ramos

**Affiliations:** 1 Universidade Estadual de Maringá, Departamento de Odontologia (Maringá/PR, Brazil).

**Keywords:** Orthodontic retainers, Gingivitis, Orthodontics, corrective, Periodontics

## Abstract

**Introduction:**

bonded fixed retainers are often used to stabilize the results obtained with the orthodontic treatment. It is important that they do not prejudice dental health, as they will be used for a long period.

**Objective:**

The purpose of the present study was to compare periodontal indexes between two types of bonded fixed retainers, conventional 3 x 3 plain retainer (0.8-mm orthodontic wire, bonded to the canines only) and a manufactured braided retainer (0.2 x 0.7-mm stainless steel wire, bonded to all anterior teeth) after use.

**Methods:**

a test group of 15 volunteers (aged from 18 to 25 years) used both the conventional retainer and braided retainer for six months. A randomized longitudinal study design, with a two week washout interval, was applied. The dental plaque index, gingival index and dental calculus index were evaluated. Furthermore, the calculus accumulated along the retainer wire was measured and all patients answered a questionnaire about the use, acceptance and comfort of both types of retainers.

**Results:**

the scores for plaque and gingival indexes were higher for the braided retainer (*p*< 0.05) on the lingual and proximal surfaces. The same occurred with the calculus index on the lingual surfaces (*p*< 0.05). The calculus index along wire was higher for the braided retainer (*p*< 0.05). All patients preferred the conventional retainer, and said that it was also more comfortable to use.

**Conclusion:**

it was concluded that the conventional retainer showed better periodontal indexes than the braided type.

## RESUMO

**Introdução:**

as contenções ortodônticas fixas são amplamente utilizadas após o tratamento ortodôntico, sendo fundamental que esses dispositivos não se tornem prejudiciais à saúde dentária, já que serão usados por um longo período.

**Objetivo:**

o presente estudo teve como objetivo a avaliação periodontal da região da arcada inferior, comparando as condições de acúmulo de placa e cálculo ao longo do fio e na margem gengival, em decorrência do uso da contenção convencional (fio 0,8 mm de aço inoxidável colado apenas nos caninos) ou de uma contenção pré-fabricada com fio trançado (0,2 x 0,7 mm colado em todos os dentes anteroinferiores) após exposição ao meio bucal.

**Métodos:**

participaram do estudo 15 voluntários adultos jovens (idades entre 18 e 25 anos) que utilizaram dois tipos de contenções, por seis meses cada. Foi utilizado um modelo de estudo longitudinal, randomizado, com *washout* de 15 dias. Os parâmetros periodontais utilizados foram: índice gengival, índice de placa e índice de cálculo. O cálculo acumulado ao longo da contenção foi avaliado e todos os pacientes responderam a um questionário sobre o uso, aceitação e conforto de ambos os tipos de contenção.

**Resultados:**

observou-se que o índice de placa foi maior para a contenção com fio trançado (*p*<0,05), assim como o índice gengival (*p*<0,05). O mesmo ocorreu para o índice de cálculo nas faces proximais e lingual (*p*<0,05). O índice de cálculo ao longo do fio também foi significativamente maior para a contenção com fio trançado (*p*<0,05). Em relação ao questionário aplicado, 60% dos voluntários consideraram que a contenção com fio trançado foi mais desconfortável, e todos eles preferiram a contenção convencional.

**Conclusão:**

concluiu-se que a contenção convencional apresentou melhores resultados periodontais, quando comparada à contenção pré-fabricada com fio trançado.

## INTRODUCTION

Orthodontic retainers are widely used after concluding orthodontic treatment, and are indicated to avoid crowding of the mandibular anterior teeth.^1^ ^-^ ^4^

The recommendation is that orthodontic retainers should remain in place for a long period, provided that they do not compromise periodontal health.^1^ ^-^ ^5^ However, the continuous presence of the retainer wires creates areas that are difficult to clean, favoring plaque formation and impaction of food debris.^1^ This situation may result in the development of carious lesions and calculus formation, and induce gingival inflammation and periodontal disease.^6^ Over time, these factors may lead to the loss of adjacent soft and hard tissues.^1^

Fixed retainers on mandibular anterior teeth require greater cooperation by the patient, and various designs have been proposed for this purpose, to facilitate this daily task.^2^ ^,^ ^7^ ^,^ ^8^ Nevertheless, apparently retainers bonded to mandibular anterior teeth have presented worst periodontal indexes than those observed for retainers bonded to the canines only.^9^ ^-^ ^14^ However, when there is excessive misalignment of the incisors before orthodontic treatment, it appears to be rational to stabilize them individually, considering the probable dissatisfaction of patients in case of short term instability.^2^ ^,^ ^4^ ^,^ ^7^ ^,^ ^8^ ^,^ ^15^ ^,^ ^16^ Another alternative is to inform the patient about the possibility of instability in the area, and adopt classical retainers bonded to the canines only, and if any alteration should occur, proceed with localized correction, followed by new stabilization.^11^

The use of the prefabricated Ortho-FlexTech^®^ (Reliance Orthodontic Products, USA) 3 x 3 braided retainer, bonded to all teeth, is indicated for mandibular anterior regions; and the 2 x 2 type, for maxillary regions. However, up to now, no study has been conducted demonstrating its behavior regarding the periodontal indexes.

Thus, the aim of this study was to compare the following conditions: plaque accumulation along the wire and on the gingival margin; periodontal conditions resulting from the use of conventional and Ortho-FlexTech^®^ 3 x 3 retainers. 

## MATERIAL AND METHODS

Fifteen volunteers participated in this study, which were submitted to anamnesis and initial clinical oral exam. The inclusion criteria were as follows: present good alignment of the mandibular anterior teeth; age range from 18 to 25 years; submitted to previous orthodontic treatment. The exclusion criteria were: being under orthodontic treatment; or having severe crowding of the mandibular anterior teeth. 

The volunteers received a Term of Free and Informed Consent, in accordance with the Guidelines and Regulatory Rules of the National Health Council (Resolution No. 196/96). The study began after being approved by the human research ethics committee of State University of Maringá (CAAE: 31435114.9.0000.0104). 

The study presented the following stages in the experimental design:

1. Baseline - scaling and dental prophylaxis 15 days before starting use of the retainer. On day zero, the periodontal indexes had to be normal.
  a) Use of conventional retainer/Ortho-FlexTech - for 6 months. Readout of indexes on conclusion.
2. Washout - after removal of the first retainer used, removal of residual resin, dental polishing, and waiting period of 15 days, for normalization of the indexes.
  b) Use of Ortho-FlexTech retainer/conventional - for 6 months. Readout of indexes on conclusion.

**Figure 1:**
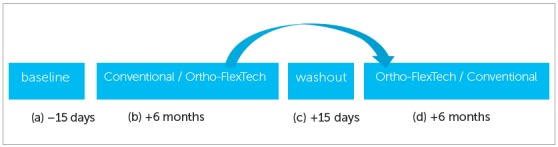
Research flow diagram: (a) Baseline - scaling and dental prophylaxis 15 days before starting use of the retainer. On day zero, the periodontal indexes had to be normal. (b) Use of conventional retainer / OrthoFlex Tech - for 6 months. Readout of indexes on conclusion. (c) Washout - after removal of the first retainer used, removal of residual resin, dental polishing, and waiting period of 15 days for normalization of the indexes. (d) Use of OrthoFlex Tech / conventional retainer - for 6 months. Readout of indexes on conclusion.

After Baseline, the volunteers used both types of retainers during the experimental period and each retainer remained for 6 months in the oral environment, with a 15-day interval between the use of the retainers, for coronal-radicular scaling, dental prophylaxis and oral hygiene instruction (washout). During the first semester, eight patients used the conventional retainer, and seven used the Ortho-FlexTech retainer. After readout of the indexes at the end of the semester and the washout interval, the volunteers who had used the conventional retainer began to use the Ortho-FlexTech, and vice-versa (Fig 1).

At the end of each semester, the indexes were read by a single experienced examiner. After the period of 6 months of using each retainer, periodontal evaluation of the mandibular anterior teeth was performed in three areas - two proximal and one lingual -, by means of the dental plaque and dental calculus indexes. In addition, the calculus on the retention wire was measured. The same previously calibrated examiner made all the evaluations. 

On conclusion of each stage of the study, the volunteers answered a questionnaire (Table 1) to evaluate the two types of retainers in terms of comfort, ease of cleaning, and acceptance by the volunteer.

**Table 1:** Questionnaire to evaluate the two types of retainers in terms of comfort, ease of cleaning, and acceptance by the volunteer.

	Conventional retainer	Ortho-FlexTech retainer
Comfort in use		
Better hygiene		
Necessary use the dental floss		
Preference in retention type		

• Respond with an X marking the preferred retainer in each case.

**Figure 2:**
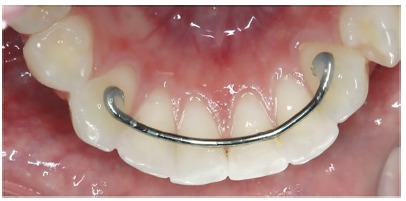
Conventional retainer bonded to mandibular canines.

**Figure 3:**
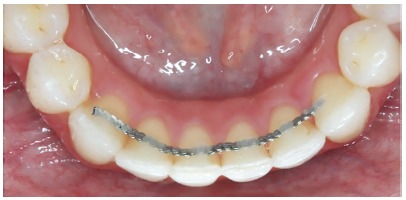
Ortho-FlexTech retainer bonded to all the mandibular anterior teeth.

The retainers were fabricated by a single orthodontist after obtaining the plaster cast of each volunteer, and were bonded to the teeth by a single experienced operator. The conventional retainers were fabricated with 0.8-mm orthodontic archwire (Morelli, Sorocaba, Brazil), and were fixed to the mandibular canines, close to the incisal middle third of the lingual surface (Fig 2). To aid bonding, a length of dental floss was folded in half and passed through the interproximal region of the central and lateral incisors on both sides. LCR composite resin (Reliance Orthodontic Products, Inc., USA) was used for bonding. The Ortho-FlexTech (Reliance Orthodontic Products, Inc., USA) retainer, prefabricated with 0.2 x 0.7mm stainless steel wire, was also fixed on all the mandibular anterior teeth, from canine to canine, close to the incisal middle third on the lingual surface (Fig 3). The bonding process was the same as that adopted for conventional retainers, with the use of the same resin (LCR, Reliance Orthodontic Products, Inc., USA).

Considering that all the variables were of the qualitative ordinal type, the Paired Wilcoxon Test was selected, at a level of significance of 5%.

## RESULTS

Table 2 presents the frequency and percentage of plaque scores per tooth surface, when the conventional and Ortho-FlexTech^®^ retainers were used. There was greater plaque accumulation when the Ortho-FlexTech^®^ retainer was used, and the results showed statistically higher values for the proximal and lingual surfaces. 

**Table 2:** Frequency and percentage of each plaque index score for each tooth surface between the Conventional and Ortho-FlexTech retainers, followed by the P value for each surface

Plaque Index			
	Conventional n (%)	Ortho-FlexTech n (%)	P-value
Mesial			
0	1 (6.7)	1 (6.7)	0.03
1	9 (60.0)	4 (26.7)	
2	5 (33.3)	8 (53.3)	
3	0 (0.0)	2 (13.3)	
Distal			
0	1 (6.7)	1 (6.7)	0.03
1	9 (60.0)	4 (26.7)	
2	5 (33.3)	8 (53.3)	
3	0 (0.0)	2 (13.3)	
Vestibular			
0	12 (80)	6 (40)	0.01
1	3 (20)	9 (60)	
2	0 (0.0)	0 (0.0)	
3	0 (0.0)	0 (0.0)	
Lingual			
0	1 (6.7)	0 (0.0)	0.001
1	9 (60.0)	2 (13.3)	
2	5 (33.3)	10 (66.7)	
3	0 (0.0)	3 (20.0)	

Table 3 presents the frequency and percentage regarding the dental calculus index, followed by the p-value of each surface. The data were statistically higher only for the mesial, distal and lingual surfaces, and the highest indexes were found after the use of the Ortho-FlexTech^®^ retainer. 

**Table 3:** Frequency and percentage of each gingival index score for each tooth surface between the Conventional and Ortho-FlexTech retainers, followed by the P value for each surface.

Gingival Index			
	Conventional n (%)	Ortho-FlexTech n (%)	P-value
Mesial			
0	5 (33.3)	2 (13.3)	0.02
1	9 (60.0)	8 (53.3)	
2	1 (6.7)	5 (33.3)	
3	0 (0.0)	0 (0.0)	
Distal			
0	5 (33.3)	2 (13.3)	0.01
1	10 (66.7)	8 (53.3)	
2	0 (0.0)	5 (33.3)	
3	0 (0.0)	0 (0.0)	
Lingual			
0	4 (26.7)	0 (0.0)	0.008
1	9 (60.0)	6 (40.0)	
2	2 (13.3)	9 (60.0)	
3	0 (0.0)	0 (00.0)	

The frequency and percentage of the gingival index and p-value for each surface are presented in Table 4. There was no difference between the retainers for the vestibular surface. Only the proximal and lingual surfaces presented statistically lower values for the conventional retainer.

**Table 4:** Frequency and percentage of each calculus index score for each tooth surface between the conventional and Ortho-FlexTech retainers, followed by the p-value for each surface.

Calculus index			
	Conventional n (%)	Ortho-FlexTech n (%)	P-value
Mesial			
0	5 (33.3)	1 (6.7)	0.02
1	8 (53.3)	7 (46.7)	
2	2 (13.3)	6 (40.0)	
3	0 (0.0)	1 (6.7)	
Distal			
0	5 (33.3)	1 (6.7)	0.01
1	9 (60.0)	8 (53.3)	
2	1 (6.7)	5 (33.3)	
3	0 (0.0)	2 (6.7)	
Lingual			
0	5 (33.3)	0 (0.0)	0.001
1	10 (66.7)	5 (33.3)	
2	0 (0.0)	10 (66.7)	
3	0 (0.0)	0 (00.0)	

The results of the calculus index along the wire are shown in Table 5. There was greater calculus accumulation along the wire in the Ortho-FlexTech retainer, and this difference was statistically significant in comparison with the conventional retainer values.

**Table 5:** Frequency and percentage of each calculus index score along the wire for the conventional and Ortho-FlexTech retainers, followed by the p-value.

Calculus index along the wire			
	Conventional n (%)	Ortho-FlexTech n (%)	P-value
0	10 (66.7)	2 (13.3)	0.005
1	5 (33.3)	8 (53.3)	
2	0 (0.0)	5 (33.3)	
3	0 (0.0)	0 (0.0)	

The results of the questionnaire are presented in Table 6. Of the volunteers, 40% found the Ortho-FlexTech^®^ retainer to be more uncomfortable; 100% of the volunteers affirmed that they were better able to clean the appliance during use of the conventional retainer. Relative to the need to use dental floss, 100% affirmed this was necessary for the Ortho-FlexTech retainer, while no volunteer pointed out this need for the conventional retainer. With respect to the preferred type of retainer, all the volunteers opted for the conventional retainer.

**Table 6:** Results of questionnaire applied to volunteers.

	Conventional retainer	Ortho-FlexTech retainer
Comfort in use	60%	40%
Better cleaning	100%	0%
Need to use dental floss	0%	100%
Preference for type of retainer	100%	0%

## DISCUSSION

Fixed retainers are widely used after orthodontic treatment, due to the known instability of the mandibular anterior region.^1^ ^-^ ^4^ However, difficulties with cleaning these retainers may lead to calculus accumulating along the wire and in proximal areas, generating periodontal impact.^1^ Eventually, retainers may be removable, facilitating cleaning of the area.^12^ However, they will be dependent on patient’s cooperation for a long period. It has been suggested that the stability of the mandibular interincisor region may only be guaranteed if retainers are maintained throughout life.^13^ ^,^ ^14^ The need for maintaining the intercanine distance is emphasized, as it undergoes changes with age, irrespective of orthodontic treatment. The question remains whether the retainers should be bonded to all the incisors, or whether it would be acceptable for them to be bonded only to the canines.

The present study revealed that there was greater plaque accumulation on the Ortho-FlexTech^®^ retainer, in comparison with that on the proximal and lingual surfaces of the flat conventional retainer. In the same way as for the plaque index, the mesial, distal and lingual surface of the gingival index presented statistically lower values for the conventional retainer, in comparison with the Ortho-FlexTech type (Table 2). Therefore, as already known, the larger the plaque accumulation, the greater the gingival inflammation.^6^ These results corroborate those of other studies.^9^ ^,^ ^10^ ^,^ ^15^ ^,^ ^16^ When the retainers are bonded to all the teeth from canine to canine, they create areas that are more difficult to clean, and deficient cleaning leads to worse consequences than slight misalignment of the area. Apart from this eventual periodontal compromise, unexpected effects have been reported with retainers bonded to all the incisors, such a root torque and gingival recessions, putting the health of the teeth at risk.^17^ ^-^ ^20^

The index of calculus along the gingival margin showed a statistically higher value for the Ortho-FlexTech^®^ retainer, in comparison with the conventional retainer, both on the proximal and lingual surfaces (Table 3). This result corroborated the indexes previously evaluated, taking into consideration that the constant presence of dental biofilm normally undergoes a process of mineralization or calcification, forming dental calculus, which - in the same way as bacterial plaque - may be above (supragingival) or below (subgingival) the gingival margin.^21^ ^,^ ^22^ There was no statistically significant difference for the vestibular surfaces relative to calculus accumulation. 

The index of calculus along the wire also demonstrated greater calculus accumulation for the Ortho-FlexTech^®^ retainer (Table 4). In addition to the difficulty of cleaning, due to the presence of resin on all the incisors, the Ortho-FlexTech is fabricated with braided wire, presenting areas that favor biofilm retention along the wire.

In spite of the increase in plaque and calculus, and the increase in local inflammatory biomarkers,^16^ in general, no bone loss related to fixed retainers in the mandibular anterior area was reported after 10 years.^23^ However, apparently this risk evaluation must be individualized.^24^

As regards the questionnaire applied to all the volunteers (Table 5), 60% declared that the conventional retainer was more comfortable, while 40% elected the Ortho-FlexTech^®^ type. The volunteers reported that the roughness of the braided retainer was more perceivable by the tongue. All the volunteers affirmed the need to use dental floss in the Ortho-FlexTech retainer, demanding more time for cleaning it. However, all related that it was possible to perform complete cleaning, with dental floss reaching up to the gingival sulcus, during the use of the latter. These reports may be justified by the fact that the volunteers were Dental students, and had knowledge of the importance of complete oral hygiene. Therefore, the conventional retainer was chosen as being the one that presented better cleaning, since it was not necessary to use dental floss for interproximal cleaning and there was no resin on all the anterior teeth. 

From the point of view of stabilizing orthodontic treatment, it may be imperative to use the fixed retainer on one segment of teeth. A retainer bonded to all the teeth for splinting after orthodontic treatment has been recommended for cases with accentuated bone loss, due to the loss of primary stability,^3^ and also for cases in which crowding of the incisors was very accentuated at the time of pretreatment.^3^ ^,^ ^25^ ^-^ ^29^ In these cases, frequent supervision is recommended, both for checking bond stability, and for controlling oral hygiene in the area.

Therefore, it appears to be reasonable to recommend bonding to all the anterior teeth only when there is specific orthodontic need or splinting is necessary.

## CONCLUSION

Based on the results obtained during the study, conventional retainers presented better periodontal results than the Ortho-FlexTech^®^ retainers. 
